# Morphological and molecular characterization of *Pungentus sufiyanensis* n. sp. and additional data on *P. engadinensis* (Altherr, 1950) Altherr, 1952 (Dorylaimida: Nordiidae) from northwest of Iran

**DOI:** 10.21307/jofnem-2020-030

**Published:** 2020-04-28

**Authors:** Nasir Vazifeh, Gholamreza Niknam, Habibeh Jabbari, Reyes Peña-Santiago

**Affiliations:** 1 Department of Plant Protection, Faculty of Agriculture, University of Tabriz, Tabriz, Iran; 2 Department of Plant Protection, Faculty of Agriculture, University of Maragheh, Maragheh, Iran; 3 Departamento de Biología Animal, Biología Vegetal y Ecología, Universidad de Jaén, Campus ‘Las Lagunillas’ s/n, Edificio B3, 23071-Jaén, Spain

**Keywords:** D2-D3, Description, Molecular analysis, Morphology, Morphometrics, Taxonomy

## Abstract

Two species of the genus *Pungentus*, one new and one known, collected in natural vegetation and cultivated soils in northwest of Iran, are studied. *Pungentus sufiyanensis* n. sp. is characterized by its 1.22 to 1.57 mm long body, offset lip region by a constriction and 7 to 9 μm broad, 18 to 21 μm long odontostyle, 304 to 348 μm long neck, 133 to 161 μm long esophageal expansion, mono-opisthodelphic female genital system without anterior uterine sac, slightly backward directed vagina, absence of *pars refringens vaginae*, *V* = 47−54, rounded-conoid caudal region (17.5–23 μm, *c* = 65–84, *c*´ = 0.7–1) with saccate bodies, and the absence of male. Molecular analysis, based on D2-D3 expansion segments of the 28S rDNA (LSU), confirms the monophyly of the family Nordiidae and suggests the monophyly of the genus *Pungentus*, with the new species forming a clade with other Iranian species. New data are presented for six Iranian populations of *P. engadinensis*, and an updated key for the identification of *Pungentus* species is also provided.

The genus *Pungentus* is an interesting dorylaimid genus, often found in forest habitats of the Northern Hemisphere, and with very restricted presence in southern territories. Its taxonomy was updated by Álvarez-Ortega and Peña-Santiago (2014), who listed 16 valid species and other four *inquirendae* or *incertae sedis* and provided a key to their identification as well a compendium of their main morphometrics.

Available information about *Pungentus* species from Iran is very limited. Solouki et al. (2010) recorded *P. engadinensis* (Altherr, 1950) Altherr, 1952 and *P. silvestris* (de Man, 1912) Coomans and Geraert (1962) in [Uremia (West Azarbaijan) and Marand (East Azarbaijan) provinces, respectively], whereas, very recently, Heydari et al. (2019) described a new species, *P. azarbaijanensis*, associated with grass in West Azarbaijan, and *P. engadinensis* in several locations of the country.

Several *Pungentus* populations were collected in the course of a nematological survey conducted in natural and cultivated soils of northwest Iran (East–West Azarbaijan and Kurdistan provinces) to explore the dorylaimid diversity of this region. Their study revealed that they belonged to one new and one known species. The objective of this work was to report *Pungentus sufiyanensis* n. sp. using morphology, morphometric, and molecular methods and provide new data about *P. engadinensis.*

## Materials and methods

### Extraction and processing of nematodes

Soil samples were collected from the rhizosphere of several crops and orchards of East–West Azarbaijan and Kurdistan provinces, northwest Iran, during the period 2010–2017. Nematodes were extracted following the protocols by Jenkins (1964) and Whitehead and Heming (1965), transferred to anhydrous glycerin according to De Grisse (1969), and mounted on glass slides for handling.

### Light microscopy

Mounted specimens were observed under an Olympus BX 41 light microscope equipped with a drawing tube and a DP50 digital camera attached to it. Morphometrics include Demanian indices and the usual measurements and ratios. Line illustrations were prepared using CorelDRAW^®^ software version 12. Microphotographs were edited using Adobe^®^ Photoshop^®^ CS software.

### DNA extraction, PCR and sequencing

For the molecular study of the new species, DNA samples were extracted from a live adult nematode, hand-picked, and placed on a clean slide containing a drop of distilled water or worm lysis buffer (WLB) and crushed by a sterilized scalpel. Then, the suspension was transferred to an Eppendorf tube containing 25.65 μl ddH2O, 2.85 μl 10 × PCR buffer and 1.5 μl proteinase K (600 μg/ml) (Promega, Benelux, the Netherlands). The tubes were incubated at −80°C (1 h), 65°C (1 h) and 95°C (15 min). The extracted DNA was stored at −20°C until use. The D2-D3 domains of the 28S rDNA were amplified with forward primer D2A (5´-ACAAGTACCGTGAGGGAAAGTTG-3´) and reverse primer D3B (5´-TCGGAAGGAACCAGCTACTA-3´) (Nunn, 1992). In total, 25 μl PCR reaction mixture was prepared constituting of 10 μl ddH_2_O, 12.5 μl master mix (Ampliqon, Denmark), 0.75 μl of each forward and reverse primers, and 1 μl of DNA template. PCR was carried out using a BIO RAD thermocycler machine in accordance with Archidona-Yuste et al. (2016). PCR cycle conditions were as follows: denaturation at 94°C for 2 min, 35 cycles of denaturation at 94°C for 30 s, annealing of primers at 55°C for 45 s and extension at 72°C for 3 min followed by a final elongation step at 72°C for 10 min. The purified PCR products were sent for sequencing to Bioneer Company, South Korea. The newly obtained sequences of *P. sufiyanensis* n. sp. were deposited in the GenBank database under accession number MN855359 as indicated on the phylogenetic tree of Table 2.

**Table 1.** Morphometric data for *Pungentus sufiyanensi*s n. sp.

	Sufiyan population			
Locality	Female		Bokan population	Marand population
Characters	Holotype	Paratypes	Female	Female
n	–	7	8	2
L	1.49	1.53 ± 0.08	1.41 ± 0.09	1.27 ± 0.07
		(1.40–1.57)	(1.33–1.54)	(1.22–1.33)
a	45	42.0 ± 3.3	47.0 ± 2.8	46.0 ± 2.3
		(40.0–47.0)	(42.0–50.0)	(43.0–49.0)
b	4.4	4.5 ± 0.1	4.4 ± 0.3	3.9 ± 0.7
		(4.3–4.8)	(4.0–5.0)	(3.9–4.0)
c	68	75.0 ± 6.1	70.0 ± 4.7	68.0 ± 1.4
		(65.0–84.0)	(66.0–75.0)	(67.0–70.0)
c´	1	0.8 ± 0.01	0.9 ± 0.07	0.9 ± 0.01
		(0.7–1.0)	(0.8–1.0)	(0.8–1.0)
V	50	49.0 ± 1.0	49.0 ± 1.0	51.0 ± 2.3
		(47.0–50.0)	(48.0–51.0)	(49.0–54.0)
Lip region diam.	8	8.4 ± 0.3	8.2 ± 0.6	8.0 ± 0.5
		(8.0–9.0)	(7.5–9.0)	(7.0–9.0)
Odontostyle length	20	19.0 ± 0.7	19.0 ± 1.4	20.0 ± 0.4
		(18.0–20.0)	(18.0–20.5)	(19.0–21.0)
Odontophore length	18	17.0 ± 0.8	16.5 ± 0.2	17.0 ± 1.8
		(15.0–18.0)	(16.0–17.0)	(16.0–19.0)
Guiding ring from ant. end	13	13.0 ± 0.5	13.5 ± 0.0	14.0 ± 0.0
		(12.0–14.0)	(13.5)	(14.0)
Neck length	325	335 ± 7	340 ± 15	315 ± 10
		(325–348)	(304–356)	(309–328)
Phar. expansion length	148	150.0 ± 2.8	149.0 ± 5.2	138.0 ± 4.7
		(147.0–155.0)	(138.0–161.0)	(133.0–144.0)
Body diam. at neck base	30	33.0 ± 1.9	29.0 ± 1.8	26.0 ± 0.0
mid-body anus		(30.0–38.0)	(27.0–31.0)	(26.0)
	32	35.0 ± 1.6	29.0 ± 0.4	27.5 ± 0.3
		(32.0–38.0)	(28.0–30.0)	(27.0–28.0)
	20	23.0 ± 2.6	19.5 ± 1.1	20.0 ± 0.6
		(20.0–26.0)	(18.0–21.0)	(19.0–21.0)
Prerectum length	75	71 ± 11	67.0 ± 3.1	83.0 ± 2.0
		(57–90)	(62.0–73.0)	(82.0–85.0)
Rectum length	18	19.0 ± 1.8	20.0 ± 1.2	20.5 ± 0.4
		(18.0–23.0)	(19.0–22.0)	(20.0–21.0)
Tail length	21	20.0 ± 1.8	19.0 ± 2.0	18.5 ± 0.2
		(18.0–23.0)	(17.5–23.0)	(18.0–19.0)

**Note:** All measurements are in μm (except L, in mm) and in the form: mean ± SD (range).

**Table 2.** Nematode species, locality, associated host and sequences used in this study.

Species	Locality	Host-plant	Accession number
*Enchodelus* cf *longispiculus*	Gorgan province, Iran	–	KP190119
*Enchodelus* sp	Hamedan province, Iran	–	KP190120
*Enchodelus* sp	–	–	EF207240
*Enchodelus macrodorus*	–	–	AY593054
*Enchodeloides signyensis*			KY881719
*Enchodorus dolichurus*	–	–	KR184124
*Enchodorus dolichurus*	–	–	KR184125
*Enchodorus yeatsi*	Andimeshk, Khuzestan province, Iran	Mosses in a natural region	KX691911
*Heterodorus youbertghostai*	Sabalan mountains, Iran	Grasslands	KR184127
*Heterodorus youbertghostai*	Arasbaran forests, Kaleybar, East-Azarbayjan province, Iran	Grasses	KR184126
*Heterodorus brevidentatus*	Kerman, Iran	–	KP963962
*Longidorella penetrans*	–	–	HM235515
*Longidorella* cf *macramphis*	–	–	AY593042
*Paravulvus hartingii*	–	–	AY593062
*Pungentus silvestris*	–	–	AY593052
*Pungentus silvestris*	–	–	AY593053
*Pungentus engadinensis*	–	–	AY593050
*Pungentus engadinensis*	Damghan, Semnan province, Iran	Fruit trees	MH346473
*Pungentus engadinensis*	Noshahr, Mazandaran province, Iran	Forest trees	MH346474
*Pungentus monohystera*	Germany	Sediment	MF325343
*Pungentus monohystera*	Germany	Sediment	MF325344
*Pungentus azarbaijanensis*	West-Azarbaijan province, Iran	Grasses	MH346476
*Pungentus azarbaijanensis*	West-Azarbaijan province, Iran	Grasses	MH346477
*Pungentus sufiyanensis* n. sp.	Sufiyan, East-Azarbaijan province, Iran	Black cherry trees (*Prunus cerasus* L.)	MN855359
*Rhyssocolpus vinciguerrae*	Astara forests, north-western Iran	Forest trees	KP204547

### Phylogenetic analyses

The newly generated sequences were aligned with the other segments of 28S rDNA gene sequences available in GenBank using MEGA6 software (Tamura et al., 2013). *Paravulvus hartingii* (de Man, 1880) Heyns, 1968 (AY593062) as outgroup was chosen. Bayesian analysis (BI) was performed using MrBayes 3.1.2 (Ronquist and Huelsenbeck, 2003). The best fit model of DNA evolution was obtained using MrModeltest 2.3 (Nylander, 2004) with Akaike-supported model in conjunction with PAUP* v4.0b10 (Swofford, 2003). BI analysis under the general time-reversible model with invariable sites and a gamma-shaped distribution (SYM + I + G) model for the 28S rDNA gene was done. After discarding burn-in samples and evaluating convergence, the remaining samples were retained for further analyses. The topologies were used to generate a 50% majority rule consensus tree and posterior probabilities (PP) were given on appropriate clades. The tree was visualized using the program Figtree 1.4.3 v.

## Results

Systematics

**Figure 1:**
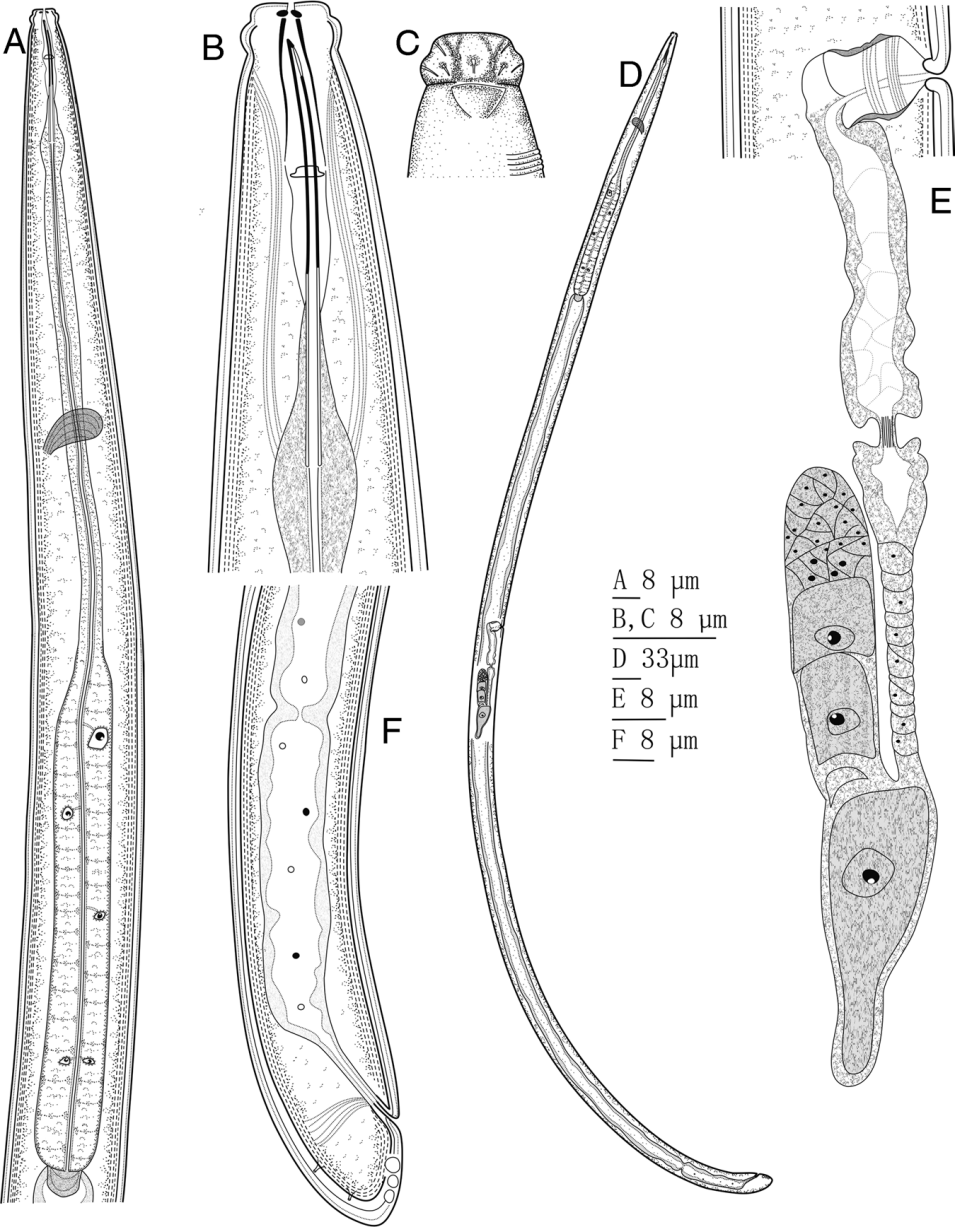
*Pungentus sufiyanensis* n. sp. (A) neck region; (B) anterior region; (C) amphidial pouch, (D) entire body, (E) genital system, (F) posterior body region.

**Figure 2:**
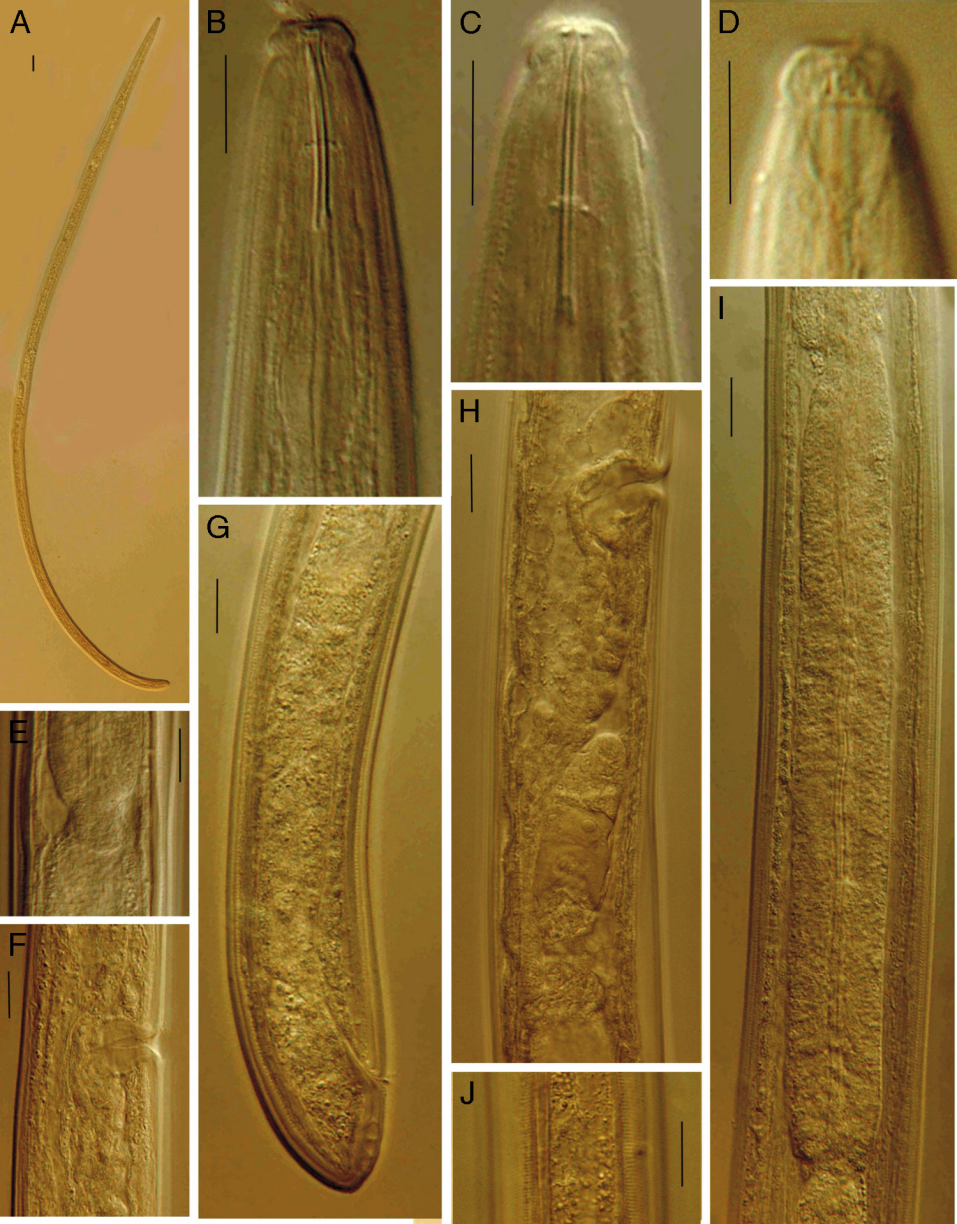
*Pungentus sufiyanensis* n. sp. (female) (A) entire, (B, C) anterior body region in lateral median view, (D) lip region in lateral surface view, (E) pharyngo-intestinal junction, (F) vagina, (G) posterior body region, (H) genital system, (I) pharyngeal expansion and pharyngo-intestinal junction, (J) lateral chord. (Scale bars: A = 32 μm; B-I = 10 μm).

*Pungentus sufiyanensis* n. sp. (Figs. 1, 2; Tables 1, 2)

### Description

Female: slender (*a* = 40-50) nematodes of medium size, 1.22 to 1.57 mm long. The body cylindrical, tapering toward both ends but more so toward the anterior extreme as the caudal region is short and rounded. Upon fixation, habitus slightly curved ventrad, to an open C-shape. Cuticle three layered, especially distinguishable at caudal region, bearing fine transverse striations, 2 to 3.5 μm thick at anterior region, 3 to 6 μm at mid-body, and 7 to 10 μm at tail. Lateral chords 8 to 11 μm thick or occupying one-fourth to one-third of mid-body diameter. The lip region is somewhat angular, offset by a weak but perceptible constriction, with nearly truncated anterior margin, 2.1 to 2.6 times as wide as height and 21 to 27% of body diameter at neck base; lips mostly amalgamated, with hardly protruding papillae. Amphidial fovea cup-shaped, opening at the level of constriction, with the aperture 4 to 5 μm long or 52 to 60% of lip region diameter. Cheilostom nearly cylindrical, 1.2 to 1.8 times as long as the lip region diameter, with visible sclerotised walls in its anterior half, and bearing four distinct, sclerotized, circumoral platelets. Odontostyle slightly arcuate dorsally, slender, well sclerotized, 2.0 to 2.5 times as long as the diameter of lip region, 1.1 to 1.4% of total body length, and aperture 2 to 3 μm long or occupying 9 to 17% its length. Guiding ring double. Odontophore rod-like, 0.8 to 1.0 times the odontostyle length. Nerve ring situated at 109 to 121 μm or 30 to 35% of the neck length from the anterior end. Pharynx entirely muscular, consisting of an anterior portion enlarging gradually into the basal expansion that is 8.4 to 13 times as long as width, 4.1 to 5.2 times as long as body diameter at neck base and occupies 40 to 45% of total neck length; gland nuclei located as follows: DN = 61−63, S_1_N_1_ = 68−70, S_1_N_2_ = 77−80, S_2_N = 89−92 according to Loof and Coomans (1970). Cardia hemispherical, almost as long as wide, 8-11 × 7-10 μm. Genital system mono-opisthodelphic, without anterior uterine sac. Genital branch well developed, 154 to 203 μm long or 9 to 16% of total body length. Ovary reflexed, 61 to 97 μm long, usually not reaching the sphincter level, with oocytes arranged first in several rows and then in a single row. Oviduct joins ovary subterminally, 48 to 62 μm or 1.1 to 2.0 times the corresponding body diameter long, consisting of a slender portion made up of prismatic cells and developed *pars dilatata* with perceptible lumen. Oviduct**–**uterus junction marked by a sphincter. Uterus a simple tube-like structure, 37 to 51 μm long or 0.9 to 1.5 times the corresponding body diameter long. Sperm in genital tract absent. Vagina slightly directed backward, extending 15 to 20 μm inwards and occupying 39 to 45% of the corresponding body diameter; *pars proximalis vaginae* 9-12 × 11-15 μm, with nearly sigmoid walls and surrounded by moderately developed, circular musculature and *pars distalis* 2 to 3.5 μm and *pars refringens vaginae* obscure in specimens examined. Vulva a nearly equatorial, transverse slit, preceded by a V-shaped depression of body surface. Prerectum 2.1 to 3.2 and rectum 0.6 to 1.0 times as long as the anal body diameter. The caudal region short, rounded-conoid, slightly more straight at the ventral side, where it bears saccate bodies; two pairs of caudal pores are present.

*Male*: unknown.

**Figure 3:**
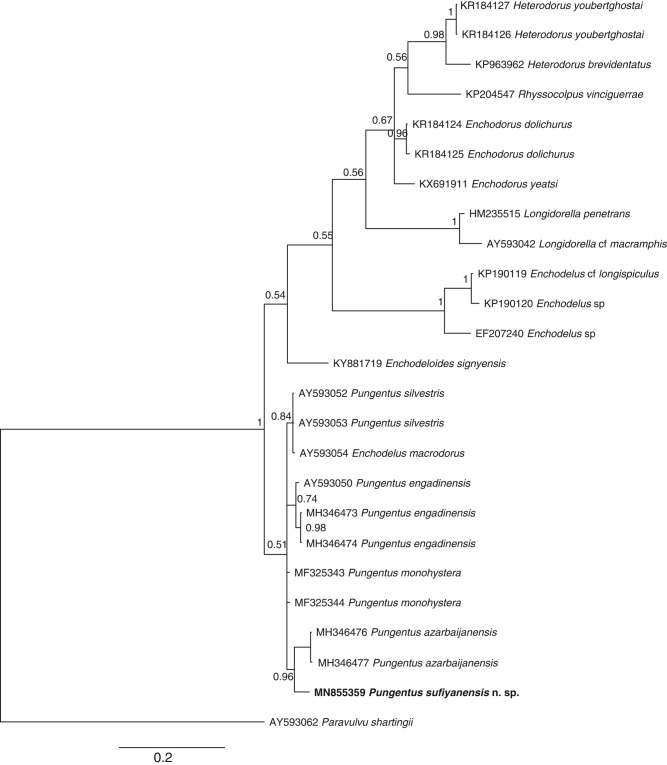
Phylogenetic tree of the *Pungentus sufiyanensis* n. sp. using D2-D3 expansion segments of the 28S rDNA gene inferred from a Bayesian analysis under SYM + I + G model (−lnL = 3,311.6086; AIC = 6,637.2173; freqA = 0.2474; freqC = 0.2381; freqG = 0.2699; freqT = 0.2446; R(a) = 1.0335; R(b) = 5.3584; R(c) = 1.8784; R(d) = 0.7817; R(e) = 8.4264; R(f) = 1.0000). Posterior probability values exceeding 50% are given on appropriate clades. Newly obtained sequence is in bold letters.

*Molecular characterization*: one sequence of the D2-D3 segment of 28S rDNA nearly 800 bp long from the new species was obtained. The results of its analysis are represented in the molecular tree of Figure 3.

*Diagnosis and relationships:* the new species is characterized by its slender (*a* = 40-50) and 1.22 to 1.57 mm long body, lip region offset by constriction and 7 to 9 μm broad, odontostyle 18 to 21 μm long, neck 304 to 348 μm long, pharynx expansion 133 to 161 μm long or 40 to 45% of total neck length, female genital system mono-opisthodelphic, without anterior uterine sac, vagina slightly directed backward, *pars refringens vaginae* absent, *V* = 47-54 and caudal region rounded-conoid (17.5-23 μm, *c* = 65-84, *c*´ = 0.7-1) with saccate bodies. Male absent.

The new species resembles *P. angulatus* Jairajpuri and Baqri, 1966 and *P. longidens* (Thorne and Swanger, 1936) Andrássy, 1986 in its mono-opisthodelphic female genital system, with the absence of prevulval sac and comparatively short odontostyle (less than 30 μm long) and caudal region (*c*-ratio more than 60). Nevertheless, it differs from *P. angulatus*, an Indian species also known to occur in Hungary (Andrássy, 2009), by having larger general size (1.22-1.57 vs 0.8-1 mm long, *n* = 22), lip region offset by a weak (vs strong) constriction, longer odontostyle (18-21 vs 14-16 μm) and neck (304-348 vs 225 μm), and relatively shorter female tail (*c’* = 0.7-1 vs 1.3) with (vs without) saccate bodies. It differs from *P. longidens*, a poorly known (but apparently close) species originally described from Spain, by its shorter odontostyle (18-21 vs 26 μm long, 2.0-2.5 times vs hardly more than thrice the lip region diameter), more posterior location of guiding ring (at appreciably more vs less than lip region diameter from the anterior end), and rounded conoid (vs short rounded to hemispheroid) female tail with (vs without) saccate bodies.

*P. sufiyanensis* n. sp. is phylogenetically related to *P. azarbaijanensis* but can be differentiated by the female genital system (mono-opisthodelphic vs didelphic-amphidelphic).

A Nblast search of the D2-D3 sequence of *P. sufiyanensis* n. sp. showed 96, 96, 99, 96, and 95% of similarity with *P. azarbaijanensis* (MH346476), *P. engadinensis* (AY593050),  *P. monohystera* (MF325343), *P. silvestris* (AY593052), and *Enchodelus macrodorus* (AY593054), respectively, with 27, 26, 2, 26, and 26 different nucleotides, respectively, too. As derived from the analysis of the new sequence herein obtained, the evolutionary relationships of the new species with other representatives of the order Dorylaimida are shown in Figure 3. The most remarkable achievement is that the new species comes close to *P. azarbaijanensis*, another Iranian species. These both species form a clade together with other *Pungentus* species, suggesting a low supported monophyly of this genus based upon currently available sequences. All the sequences of Nordiidae representatives constitute a highly supported (100%) clade, a fact that confirms the monophyly of this taxon. Leaving aside *Pungentus* sequences, the remaining ones form together a second clade, which is not well supported, within the family Nordiidae.

*Type habitat and locality:* the habitat and locality type was Northwest Iran, East-Azarbaijan province, Sufiyan, Roodghat area, Zeinabad village (GPS coordinates: N 38°17´ 30˝, E 46° 07´ 53˝, altitude 1527 m a.s.l.), where the specimens were collected from the rhizosphere of black cherry trees (*Prunus cerasus* L.).

*Other localities and habitats:* samples were collected from two locations in Northwest Iran: East-Azarbaijan province, Marand district, Kondolaj village, from the rhizosphere of almond and walnut trees; West Azarbaijan province, Bokan district, Khorasaneh area (GPS coordinates: N 36°35´ 68˝, E 46° 00´ 90˝) from the rhizosphere of natural vegetation.

*Type material:* female holotype and paratypes were deposited with the Nematode Collection of the Department of Plant Protection, Faculty of Agriculture, University of Tabriz, Tabriz, Iran. The new species binomial has been registered in the Zoobank database (zoobank.org) under the identifier B1F2B3F6-558F-4688-BFFC-0F90BD101357.

*Etymology:* the species name refers to the type locality of the new species, Sufiyan, East-Azarbaijan province, northwest of Iran.

*Pungentus engadinensis* (Altherr, 1950) Altherr, 1952.

(Fig. 4; Table 3)

**Table 3.** Morphometric data for six Iranian populations of *Pungentus engadinensis*.

Locality	Urmia population	Divandarreh population	Bokan population	Maragheh population	Sufiyan population	Basmenj population
Characters	Female	Female	Female	Female	Female	Female
n	5	6	7	7	5	6
L	0.90 ± 0.06	1.10 ± 0.01	0.99 ± 0.07	1.00 ± 0.05	0.95 ± 0.03	0.99 ± 0.03
	(0.83–1.00)	(0.91–1.22)	(0.90–1.10)	(0.90–1.10)	(0.90–0.99)	(0.96–1.06)
a	39.0 ± 2.4	40.5 ± 0.5	39.0 ± 2.3	35.5 ± 1.4	38.0 ± 1.9	34.8 ± 2.0
	(36.5–42.0)	(36.5–50.5)	(34.5–41.5)	(34.0–37.0)	(36.0–41.0)	(35.0–37.0)
b	3.7 ± 0.2	4.0 ± 0.6	3.9 ± 0.2	4.0 ± 0.2	4.0 ± 0.1	3.8 ± 0.2
	(3.5–4.5)	(3.8–5.0)	(3.5–4.0)	(4.0–4.5)	(3.9–4.2)	(3.5–4.2)
c	47.0 ± 4.6	60.5 ± 5.5	52.0 ± 0.5	60.5 ± 6.4	54.0 ± 3.4	58.6 ± 7.7
	(42.0–52.5)	(55.0–68.0)	(44.5–59.0)	(52.0–72.0)	(50.0–59.0)	(46.0–68.0)
c´	1.1 ± 0.08	0.9 ± 0.09	1.0 ± 0.01	0.0 ± 0.06	0.94 ± 0.05	0.81 ± 0.07
	(1.0–1.2)	(0.7–1.1)	(0.9–1.1)	(0.8–0.9)	(0.9–1.0)	(0.7–0.9)
V	48.0 ± 2.5	45.5 ± 0.8	46.0 ± 1.4	46.0 ± 1.3	47.0 ± 1.6	44.3 ± 3.2
	(44.0–52.0)	(44.5–47.0)	(44.0–48.0)	(44.0–47.0)	(45.0–49.0)	(41.0–49.0)
Lip region diam.	8.3 ± 0.5	10.6 ± 0.2	10.0 ± 0.6	10.0 ± 0.5	8.6 ± 0.4	8.8 ± 0.4
	(8.0–9.0)	(10.0–11.0)	(8.0–11.0)	(9.0–11.0)	(8.0–9.0)	(8.0–9.0)
Odontostyle length	15.0 ± 0.5	18.0 ± 0.5	15.3 ± 0.1	17.5 ± 0.2	16.0 ± 0.0	15.9 ± 0.9
	(14.5–16.0)	(17.5–18.5)	(14.0–17.0)	(16.0–18.0)	(16.0)	(15–17.5)
Odontophore length	19.5 ± 0.6	15.6 ± 2.1	16.6 ± 1.8	20.8 ± 1.3	14.2 ± 0.6	19.6 ± 0.9
	(19.0–20.5)	(13.0–18.5)	(14.1–19.3)	(19.0–22.0)	(13.0–15.0)	(17.0–21.0)
Guiding ring from ant. end	10.3 ± 0.5	11.2 ± 0.4	10.6 ± 0.8	10.3 ± 0.4	10.2 ± 0.4	11.3 ± 0.4
	(10.0–11.4)	(11.0–12.0)	(9.0–12.0)	(10.0–11.0)	(9.0–11.0)	(9.6–12.0)
Neck length	238 ± 13	265 ± 14	259 ± 21	302 ± 13	236 ± 11	254 ± 10
	(223–255)	(233–295)	(228–293)	(228–329)	(219–248)	(243–266)
Phar. expansion length	91.2 ± 8.3	104.2 ± 3.1	89.6 ± 9.7	108.2 ± 4.4	96.0 ± 9.1	101.0 ± 5.2
	(84.0–105.0)	(102.0–108.0)	(81.0–106.0)	(101.0–116.0)	(85.0–108.0)	(95.0–109.0)
Body diam. at neck base	26.2 ± 0.4	29.6 ± 2.7	22.2 ± 1.5	26.0 ± 0.7	23.0 ± 0.7	26.0 ± 0.9
	(25.0–27.0)	(25.0–34.0)	(21.0–24.1)	(25.0–27.0)	(22.0–24.0)	(25.0–27.0)
mid-body	27.4 ± 0.3	32.2 ± 2.0	23.3 ± 0.8	28.3 ± 0.4	24.8 ± 0.1	28.2 ± 1.3
	(27.0–28.2)	(28.0–37.0)	(22.0–25.0)	(27.0–29.1)	(24.0–25.0)	(27.0–30.0)
anus	18.3 ± 0.5	22.3 ± 2.3	18.1 ± 0.6	20.0 ± 0.7	18.0 ± 0.7	20.0 ± 1.7
	(17.0–19.0)	(20.0–25.0)	(17.0–19.0)	(19.0–21.0)	(17.0–19.0)	(19.0–22.0)
Prerectum length	72.6 ± 13.2	85.1 ± 12.4	55.2 ± 7.7	82.2 ± 2.2	40.0 ± 6.8	64.3 ± 5.1
	(52.0–94.0)	(72.0–101.0)	(50.3–64.0)	(77.0–88.0)	(30.2–50.0)	(59.0–69.0)
Rectum length	17.6 ± 0.2	20.3 ± 1.5	14.3 ± 0.5	18.1 ± 1.2	16.0 ± 2.4	22.0 ± 2.2
	(16.0–18.0)	(18.0–22.0)	(13.0–16.0)	(16.0–20.0)	(14.0–20.0)	(19.0–25.0)
Tail length	18.5 ± 0.8	17.5 ± 0.9	19.0 ± 0.7	16.7 ± 1.2	18.0 ± 0.8	17.1 ± 2.4
	(17.5–22.5)	(16.5–21.0)	(18.0–20.0)	(14.0–18.0)	(17.0–19.0)	(15.0–21.0)

**Note:** All measurements are in μm (except L, in mm) and in the form: mean ± SD (range).

**Figure 4:**
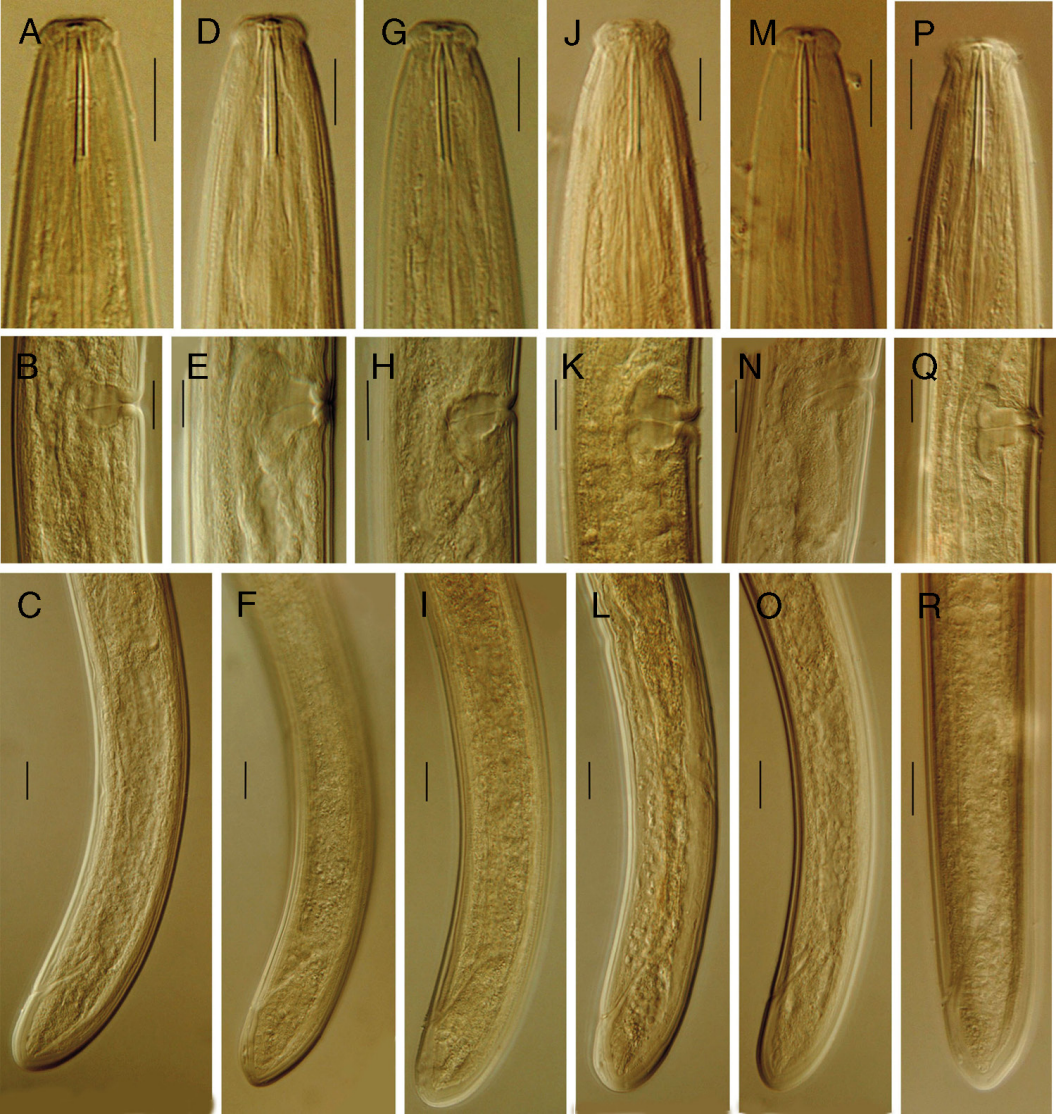
*Pungentus engadinensis* (Altherr, 1950) Altherr, 1952, Anterior region, Vaginae and Posterior body region of (A-C) Urmia; (D-F) Divandarreh; (G-I) Bokan; (J-L) Maragheh; (M-O) Sufiyan and (P-R) Basmenj populations, respectively. (Scale bars 10 μm).

*Remarks:* the six populations of this species herein examined are, morphologically and morphometrically, very similar to each other, but some minor differences have also been noted, which are regarded as intraspecific variations. Anterior uterine sac according to Andrássy (2009) and Peña-Santiago et al. (2013), varying from absent (as in our population) to present with different sizes. Thus, anterior uterine sac in Sufiyan population varied from absent to 8.5 μm long, but in all the remaining populations it was of different sizes. Saccate bodies were occasionally present (Peña-Santiago et al., 2013), and according to Heydari et al. (2019) saccate bodies were not present in their own Belgian populations and not seen in Sufiyan and Urmia populations but they were present in Divandarreh, Bokan, Maragheh, and Basmenj populations. *Pars refringens vaginae*, consisting of two small sclerotized pieces, were distinguishable in Divandarreh and Maragheh populations, but they were more inconspicuous in other populations. Vagina orientation also displays some differences: backwards directed in Bokan and Sufiyan populations and near perpendicular to body axis in other populations. Present Iranian populations of *P. engadinensis* fit very well with those previously studied by other authors (for comparative purposes, see Coomans and Geraert, 1962; Andrássy, 2009; Peña-Santiago et al., 2013; Álvarez-Ortega and Peña-Santiago, 2014; Heydari et al., 2019).

*Pungentus engadinensis* is a widely distributed species, having been recorded in Asia, Europe, and North America, where it mostly inhabits moist soils (Andrássy, 2009). In Iran, it has previously been reported (Kazemi, 2016) from the rhizosphere of vineyards in Uremia, West-Azarbaijan province; rangelands in Divandarreh, Kurdistan province; natural vegetation in Bokan, West-Azarbaijan province and Maragheh, East-Azarbaijan province; common wheat from Sufiyan, East-Azarbaijan province and Basmenj, East-Azarbaijan province, but in the form of taxonomic papers from three locations of the country reported by Heydari et al. (2019) and Solouki et al. (2010).

Key to species of the genus *Pungentus*

(Modified after Álvarez-Ortega and Peña-Santiago, 2014)
